# Association between oxidative stress and altered cholesterol metabolism in patients with Bipolar Disorder

**DOI:** 10.1192/j.eurpsy.2023.833

**Published:** 2023-07-19

**Authors:** W. Guidara, M. Messedi, M. Naifar, K. Ben Hassen, D. Bonnefont-Rousselot, F. Lamari, M. Maalej, M. Maalej, F. Makni-Ayadi

**Affiliations:** 1 Laboratory of Research “Molecular Basis of Human Diseases”, LR19ES13, Faculty of Medecine of Sfax, SFAX, Tunisia; 2 Service de Biochimie Métabolique, AP-HP. Sorbonne Université, Hôpitaux Universitaires Pitié-Salpetriére-Charles Foix, DMU BioGeM, F-75013 Paris, France, Sorbonne Université, Hôpitaux Universitaires Pitié-Salpetriére-Charles Foix, PARIS, France; 3 Psychiatry “C” department, Hedi Chaker University Hospital, SFAX, Tunisia

## Abstract

**Introduction:**

Oxidative stress is the main characteristic of several diseases including Bipolar Disorder (BD). The involvement of oxysterol derivatives has recently been reported. In this study, the involvement of oxidative stress in the alteration of cholesterol in PTB patients will be evaluated.

**Objectives:**

To assess the association of oxidative stress and oxysterol profiles in subjects with BD and compare them to healthy physical and mental controls.

**Methods:**

This is a case-control study involving subjects with BD. Selected based on DSM-5 criteria, an assessment of positive and negative symptoms was performed using the Positive and Negative Syndrome Scale (PANSS). Controls included in this study were matched to patients by age and gender. For all patients and control. Eight parameters of oxidative status were assessed: plasma ferric reducing capacity (FRAP), carbonyl proteins (PC), protein products of advanced oxidation (AOPP), reduced glutathione (GSH), total thiols, malondialdehyde (MDA ), glutathione peroxidase activity (GSH-Px) and catalase activity (CAT) analyzed by colorimetric methods. In addition, six cholesterol derivatives: oxysterols are measured by ULPC MS/MS.

**Results:**

This study included 33 patients with BD and 40 controls. Plasma GSH levels were significantly reduced in patients compared to controls (p < 0.001). Moreover, MDA, AOPP, PC and GSH-Px activity were significantly increased in patients compared to controls (p=0.005; p=0.003; p<0.001 and p=0.05, respectively). Significantly higher levels were observed for cholestane-3β, 5α, 6β-triol, 27-hydroxycholesterol (27-OHC), and cholestanol in patients with PTB. The concentration of 24(S)-hydroxycholesterol (24-OHC) was significantly lower in patients compared to controls. 25-OHC was positively and significantly correlated with CAT and GSH-Px activities (p=0.035 and p=0.010). 27-OHC was negatively and significantly correlated with MDA (p=0.014). Binary logistic regression revealed an association between the parameters: 27-OHC, 24-OHC, PC and MDA and the occurrence of PTB (OR = 1.007, 95% CI= 1.002-1.013), (OR = 0.956; 95% CI = 0.927 – 0.986), (OR = 39.925; 95% CI = 1.101 – 44.483) and (OR = 4.238; 95% CI = 1.091 – 16.466), respectively.

**Conclusions:**

Our data support the relationship between disruption of redox homeostasis and oxidation of lipids and cholesterol in BD.

**Disclosure of Interest:**

None Declared

